# Petrography and geochemistry of Paleocene-Eocene (Ewekoro) limestone, eastern Benin basin, Nigeria: implications on depositional environment and post-depositional overprint

**DOI:** 10.1016/j.heliyon.2021.e08579

**Published:** 2021-12-09

**Authors:** I.O. Adelabu, S.A. Opeloye, O.A. Oluwajana

**Affiliations:** a Department of Applied Geology, Federal University of Technology, Akure, Nigeria; b Department of Earth Sciences, Adekunle Ajasin University, Akungba-Akoko, Nigeria

**Keywords:** Ewekoro, Limestone, Stable isotope, Diagenesis, REE

## Abstract

Petrographic and geochemical studies were undertaken on the Paleocene-Eocene limestones of the Ewekoro Formation in order to infer the depositional and post-depositional imprints on the limestone. Thin section petrographic studies revealed three distinct microfacies; grainstone, packstone and wackestone, and there were variations in the depositional environments from meteoric vadose to shallow marine environments with evidences of post-depositional processes of compaction, micritization and dolomitization. The total rare earth element concentration (ƩREE) of the limestone samples ranged from 39.93 to 103.11ppm with negative Ce/Ce∗ values of 0.67–0.87 and depleted europium anomalies of 0.48–0.69. The carbon isotopic signals δ^13^C ranged from -1.4‰ to +1.9‰ V_PDB_ while the oxygen isotope δ^18^O is from -3.5‰ to -0.9‰ V_PDB_ except for a positive value of 0.5‰ observed in one limestone sample. The δ^13^C vs δ^18^O plot and Mn/Sr ratio suggested that the δ^13^C measured values are primary in nature and remain unaltered during diagenesis. The δ^18^O values showed slight alteration with negative values and indicating meteoric diagenesis and the estimated paleotemperature ranged from 19 °C to 39 °C.

## 1 Introduction

### 1.1 General statement

The evaluation of the environment of deposition and post-depositional imprints on limestone deposits requires an integration of various approaches, including standard optical petrography, cathodoluminescence, scanning electron microscopy (SEM), stable isotope analysis and measurement of rare element (REE) concentrations. Geochemistry is an essential tool in determining the depositional imprints and overprints of post-depositional processes on sediments (Murray et al., 1991; Madhavaraju and Lee, 2009; Akaegbobi and Ogungbesan, 2016; Ozkan, 2019; Patra et al., 2021). The study of the distribution of rare-earth elements (REEs) in sediments affords valuable insight into the composition of seawater and its interaction with the surrounding earth systems (Bau and Dulski, 1996). The carbon and oxygen isotopic signatures (denoted by δ^13^C and δ^18^O respectively) of limestones offer measures for the deduction of the paleoenvironment for ancient samples of different geological ages (Cuna et al., 2001; Crne et al., 2014; Kim et al., 2015). Carbon isotopes are used to infer the global organic carbon budget (Madhavaraju et al., 2013; Singh et al., 2016), while oxygen isotopes depend on the salinity and temperature of the water in which the limestones were deposited (Kim and O'Neil, 1997; Cuna et al., 2001; Singh et al., 2016). Variations in stable isotopes of carbon and oxygen of limestones may change under post-depositional processes, or may mirror the sedimentary paleoenvironment in which they were formed (Armstrong-Altrin et al., 2009; Nagarajan et al., 2013).

Previous studies on the Paleocene-Eocene Ewekoro Limestones have focussed more on its economic uses (Oyedele et al., 2016), age, stratigraphy and depositional environment (Adegoke, 1977; Omatsola and Adegoke, 1981; Elueze and Nton, 2004; Adekeye and Akande, 2007; Akinmosin and Osinowo, 2010; Akaegbobi and Ogungbesan, 2016), only a few studies (Erdtmann, 2005; Nton and Adeyemi, 2015) have considered the post-depositional overprints on limestone deposits in the eastern Benin (Dahomey) Basin.

In this work, the depositional imprints and post-depositional alterations of outcrop and core samples of Paleocene-Eocene (Ewekoro) Limestone in the eastern Benin (Dahomey) Basin was studied, using optical petrographic studies, X-ray powder diffraction (XRD) analysis, rare earth element (REE) concentrations, and carbon and oxygen stable isotopic signatures.

**Figure 1:**
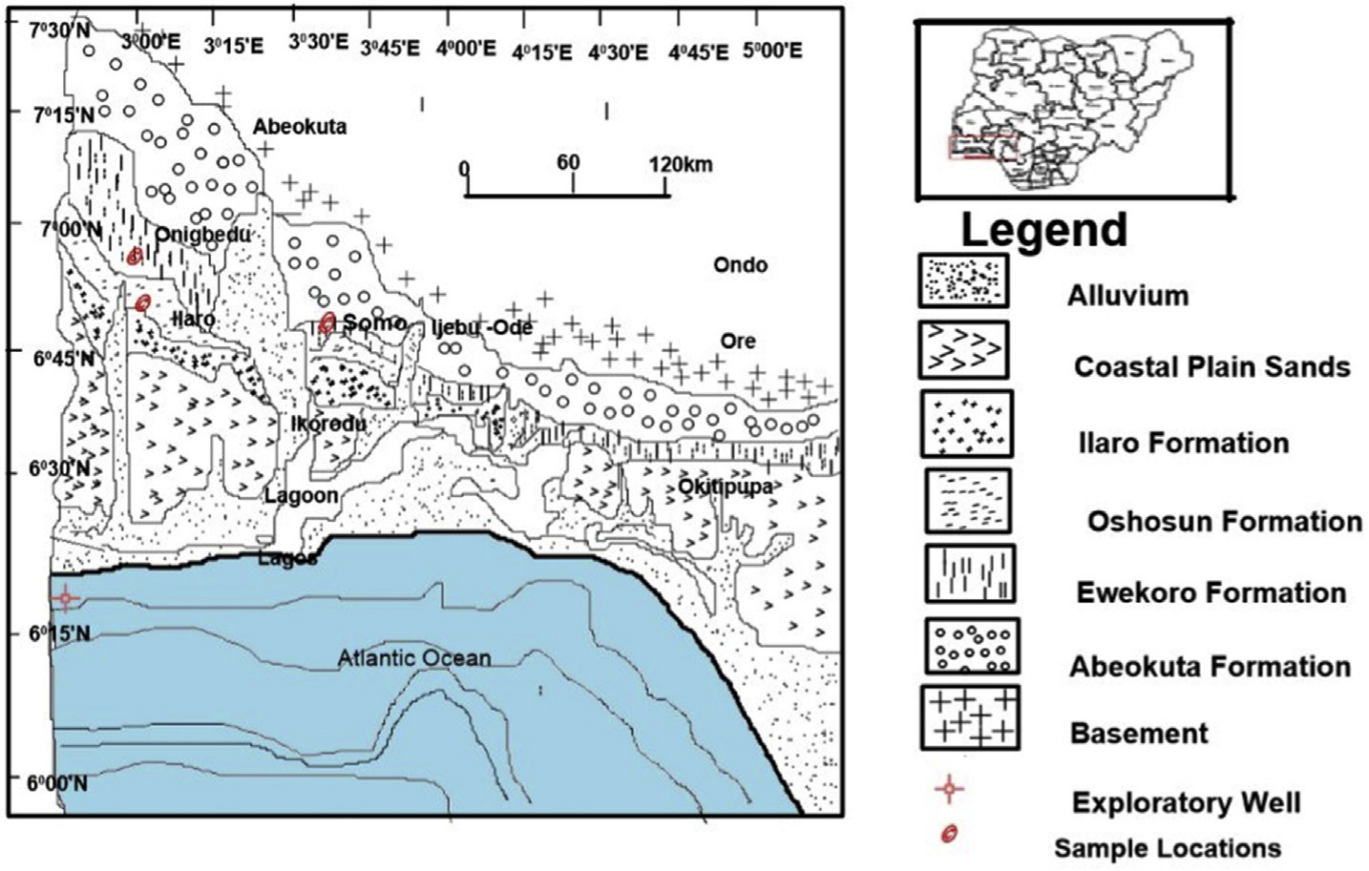
Regional geological map of the Nigerian sector of the Benin Basin showing the Cretaceous to Recent sediments, and the Sample Locations. (After Adekeye et al., 2019; Used with permission from Elsevier, Heliyon).

The exposed quarry sections of the PureChem Industries limited cement factory at Onigbedu, and an exposed section at Somo village, Ogun state Nigeria were sampled for the present study. The sampled part of the quarry is located at longitude E003^0^ 06′24.0″ and latitude N06° 57’.57.1″ and that of Somo village, was at longitude E003°31′0.012″ and latitude N06°48’ (Figure 1). The subsurface samples were acquired from the core repository of the Nigeria Geological Survey Agency (NGSA) Kaduna.

### 1.2 Geological setting

The Nigerian portion of the Benin (Dahomey) basin extends from the boundary between Nigeria and Benin Republic (in the area of the Gulf of Guinea), eastwards and terminates at the Okitipupa Ridge/Benin Hinge Line, which is the continental extension of the Chain Fracture Zone (CFZ), a major fault structure marking the western limit of Niger Delta **(**Omatsola and Adegoke, 1981; Olabode and Adekoya, 2008; Olabode and Mohammed, 2016). It is also bounded in the north by the Precambrian basement rock and the Gulf of Guinea in the south (Omatsola and Adegoke, 1981). The basin is one of a series of West African Atlantic Margin basins (marginal pull-apart/marginal sag basin) that were initiated during the period of rifting in the late Jurassic to early Cretaceous (Omatsola and Adegoke, 1981; Whiteman, 1982; Storey, 1995; Olabode, 2006; Ehinola et al., 2016).

**Figure 2:**
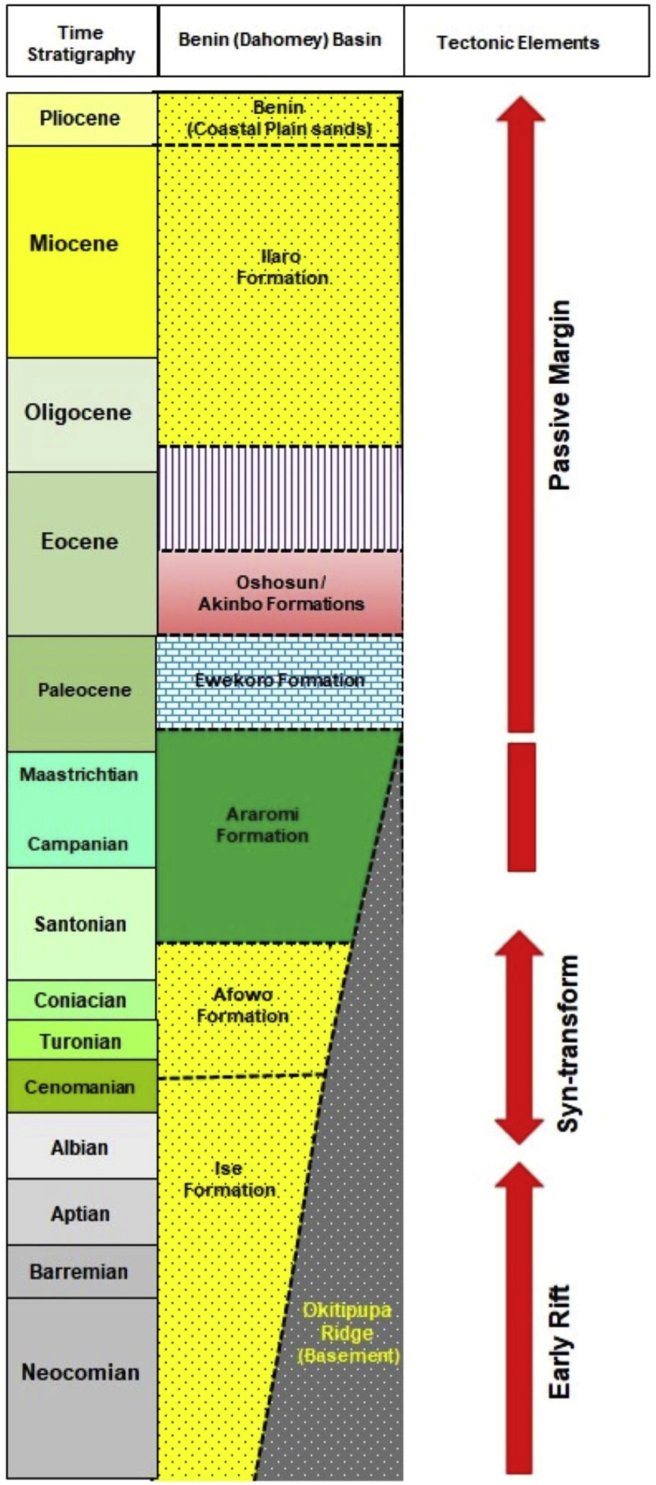
Regional simplified Cretaceous to Neogene stratigraphy of Benin Basin, southwestern Nigeria. (modified after Oluwajana et al., 2020; Used with permission from Elsevier, Journal of African Earth Sciences).

Omatsola and Adegoke (1981) reviewed the evolution and stratigraphy of the Benin basin, which was subdivided into three tectono-sedimentary stages: the pre-rift (pre-transform; Precambrian to Triassic intracratonic rocks and Jurassic to Lower Cretaceous rocks), Syn-transform (Lower Cretaceous to Late Albian rocks), passive margin (Post-transform; Cenomanian to Holocene). The Cretaceous sediments were divided into three formations; Ise, Afowo and Araromi Formations, and they are altogether referred to as the Abeokuta Group. The age of the oldest sediments (Ise Formation) is Neocomian and it is made up of conglomerates and sandstone facies, while the Araromi Formation, which is composed mainly of dark shales, ranges from Early Paleocene to Maastrichtian. Overlying the Abeokuta Group conformably is the Paleocene-Eocene Ewekoro and Akinbo Formations (Figure 2). The Ewekoro Formation is a fossiliferous shelly limestone, about 12.5 m thick, which becomes sandy in the lower parts (Nwajide, 2013). The Ewekoro Formation is overlain by an alternating sequence of shale and sandy facies with thin beds of limestones (Oshosun Formation). And this in-turn is succeeded by marine to continental Ilaro Formation. The upper section of the basin is occupied by poorly fossiliferous continental sandstone deposits and siltstone.

## 2 Sampling and methods

### 2.1 Sampling

**Figure 3:**
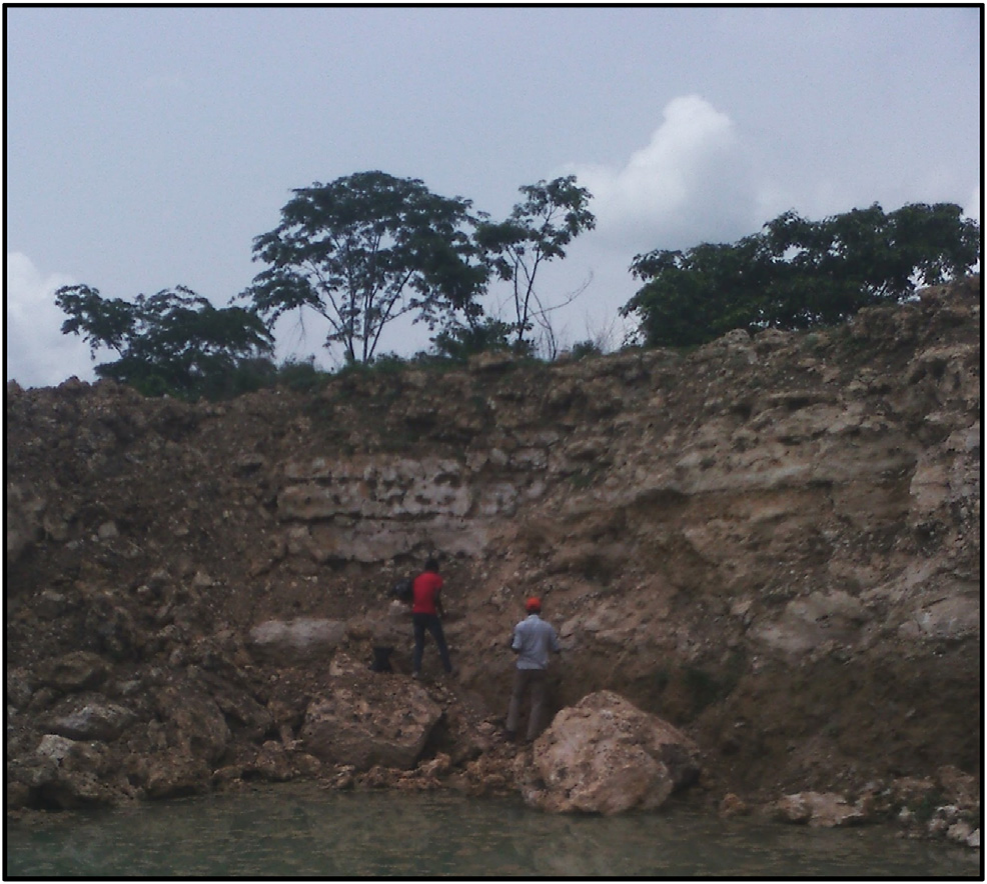
Field Photograph showing the Quarry Section at the PureChem Industries Limited.

The samples used for this study includes, five (5) limestone samples from the exposed quarry sections (Figure 3) at PureChem Cement Factory, Onigbedu (referred to as OTB samples), one (1) sample from Somo village (SOMO sample) and nine (9) subsurface limestone samples were obtained from Ewekoro (EW) and Ilaro (BH) cores from the core repository of the Nigeria Geological Survey Agency (NGSA), Kaduna, Nigeria.

### 2.2 Petrography

Fifteen thin slides of the limestone samples were prepared according to standards at the Petrology laboratory, Department of Applied Geology, Federal University of Technology, Akure. Pictures were taken using a 9MP AmScope® microscope camera. Relevant literature (Adams and Mackenzie 1998; Flugel 2010) was consulted for carbonate sediment interpretation.

### 2.3 X-ray diffraction (XRD)

X-ray powder diffraction (XRD) analyses of eleven (11) representative samples (both surface and subsurface) were carried out at the Departamento de Quimica, Laboratorio, Universidade Federal de Minas Gerais, Brazil. Powder XRD patterns were collected using a PANalytical, X'pert PRO X-ray Diffractometer.

### 2.4 Stable isotope geochemistry

The measurement of variations in the stable isotopes of carbon and oxygen of the studied fifteen (15) limestone samples were carried out at the University of Wyoming Stable Isotope Facility, USA using the Thermo Finnigan Delta Plus XP, TC LC PAL Autosampler, Thermo Finnigan GasBench II.

The ^13^C/^12^C composition of carbonates is determined by acidification of the sample with 99.99% phosphoric acid. The carbon isotopic composition is reported in per mil (‰) relative to VPDB scale, with a precision of +1.95‰. For oxygen isotope ratio composition, if the standard uncertainty is greater than 0.2‰, the unknowns are re-analysed (until the 2-sigma expanded standard uncertainty of the result is better than 0.4‰). The oxygen isotopic composition is reported in per mil (‰) relative to VPDB scale and precision of -2.2‰.

### 2.5 Rare earth elements (REE)

Rare earth element analysis of four samples OTB 4, EW 14, BH10 and SOMO were carried out at the Bureau Veristas Mineral Laboratory, Canada; using the Ultra-trace ICP-ES/MS (MA250) method. A multi-acid digestion capable of dissolving most minerals, which gives near total values for about 59 elements. A 0.25g split is heated in HNO_3_, HClO_4_ and HF to fuming and taken to dryness, the residue is then dissolved in HCl.

## 3 Results

### 3.1 Petrography

Petrographic study of the Paleocene-Eocene Ewekoro limestone allowed a microfacies classification and was described based on carbonate classification of Dunham (1962). Three microfacies types have been identified.

#### 3.1.1 Grainstone

**Figure 4:**
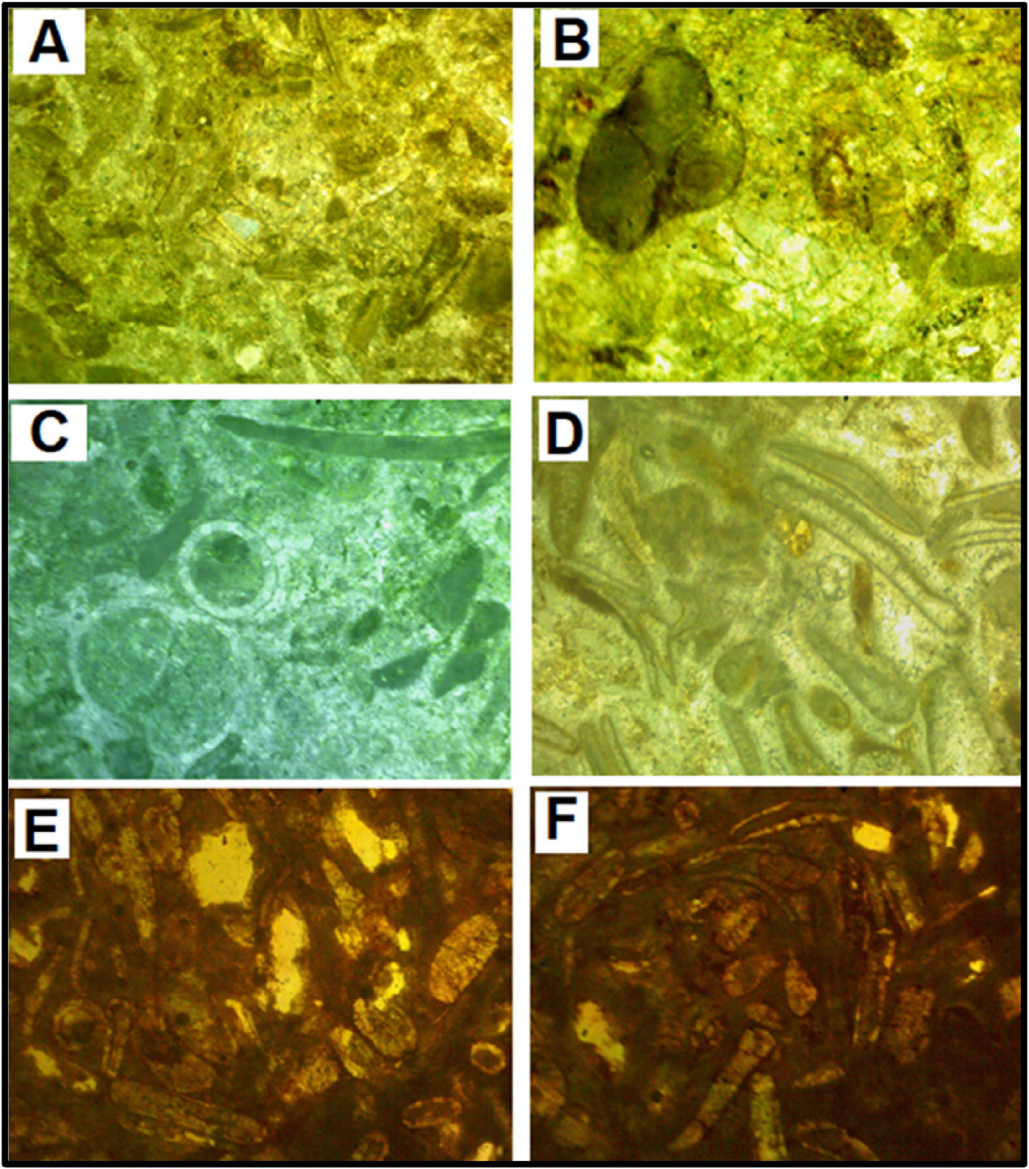
Thin Section Photomicrographs of the studied Paleocene-Eocene (Ewekoro) Limestone samples **A-C** Gastropod-foraminifera-Grainstone shows a grain supported rock with coarse grained neomorphic cement, shells/fragments of gastropods and foraminifera are common. In **4C**, a circumgranular rim cementation can be seen around the gastropod grain **D – E** Bivalve-Ooid Grainstone the grains are touching with some forming stylolites structure. Dark carbonate mud fill the intraparticle pores. **F.** Bivalve-Grainstone grain supported bivalve fragments with microcrystalline cement.

Gastropod foraminifera Grainstone **-** The gastropod shells were identified by their inward spire. Most extant gastropod shells have crossed-lamella structure and are made wholly of aragonite. In this microfacies, the gastropods observed were preserved as casts with outer layers of radial-fibrous calcite and well preserved while the inner aragonite has been dissolved and replaced by cements. The cement is seemingly coarse, mosaic and of equant sizes but some have a peloidal texture (Figure 4A-C).

Bivalve-Ooid Grainstone - The grains and the ooids are touching forming stylolites. Some of the grains have parallel contacts. The bivalves observed are fairly curved. Small sized ostracods and elliptical pelecypods were observed. The intra-particulate pores are not filled with cement. The grains have experienced some dissolution due to microbial attacks and precipitation and deposition of micritic carbonate cements in the secondary pores and forming micritic envelopes around the grains (Figure 4D-E).

Bivalve Grainstone - This molluscan grainstone microfacies encloses curved bivalve fragments with neomorphic microcrystalline spar cement (Figure 4F). The bivalve fragments are filled with precipitated spar cement with some few quartz grains, showing undulose extinction.

#### 3.1.2 Packstone

**Figure 5:**
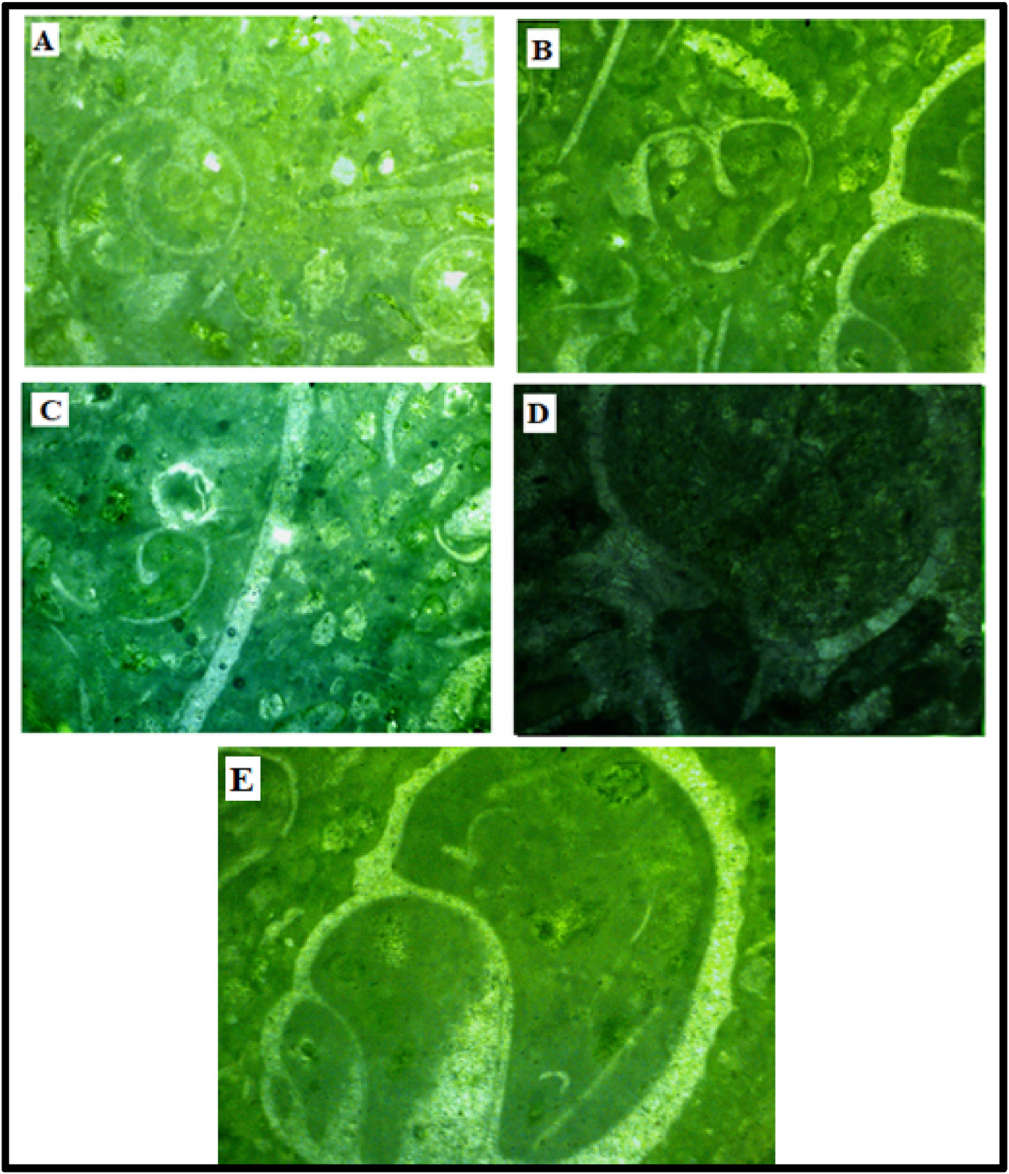
A-C. Cephalopod – Gastropod – Brachiopod Packstone cephalopods casts, strongly recrystallised gastropod moulds and fragments of brachiopods are abundant. A few rhombic crystals could also be seen in **C,** and this was interpreted as dolomite grains. **D.** close-up view of a grain with an interparticle meniscus cement, common in vadose zones. **E.** is a close-up view of gastropod showing thick rims of calcite replacing the aragonitic outer layers.

Cephalopod-Gastropod-Brachiopod Packstone - Casts of cephalopods are abundant in this microfacies, occurring along with some other molluscan fragments and brachiopods (Figures 5A-5C). Non-skeletal materials occur as concentric ooids but some have lost their nuclei. A few rhombic crystals were also identified as dolomite (Figure 5C). Meniscus cements (Figure 5D) of sparry calcite are common. This microfacies exhibits a fabric characterized by large gastropod shells with thick rims of calcite (Figure 5E), the original aragonitic shells of the gastropod has been replaced by coarse calcite. Cement consisting of pelloids, dwarf gastropods, foraminifera and other molluscs also occur in places.

#### 3.1.3 Wackestone

**Figure 6:**
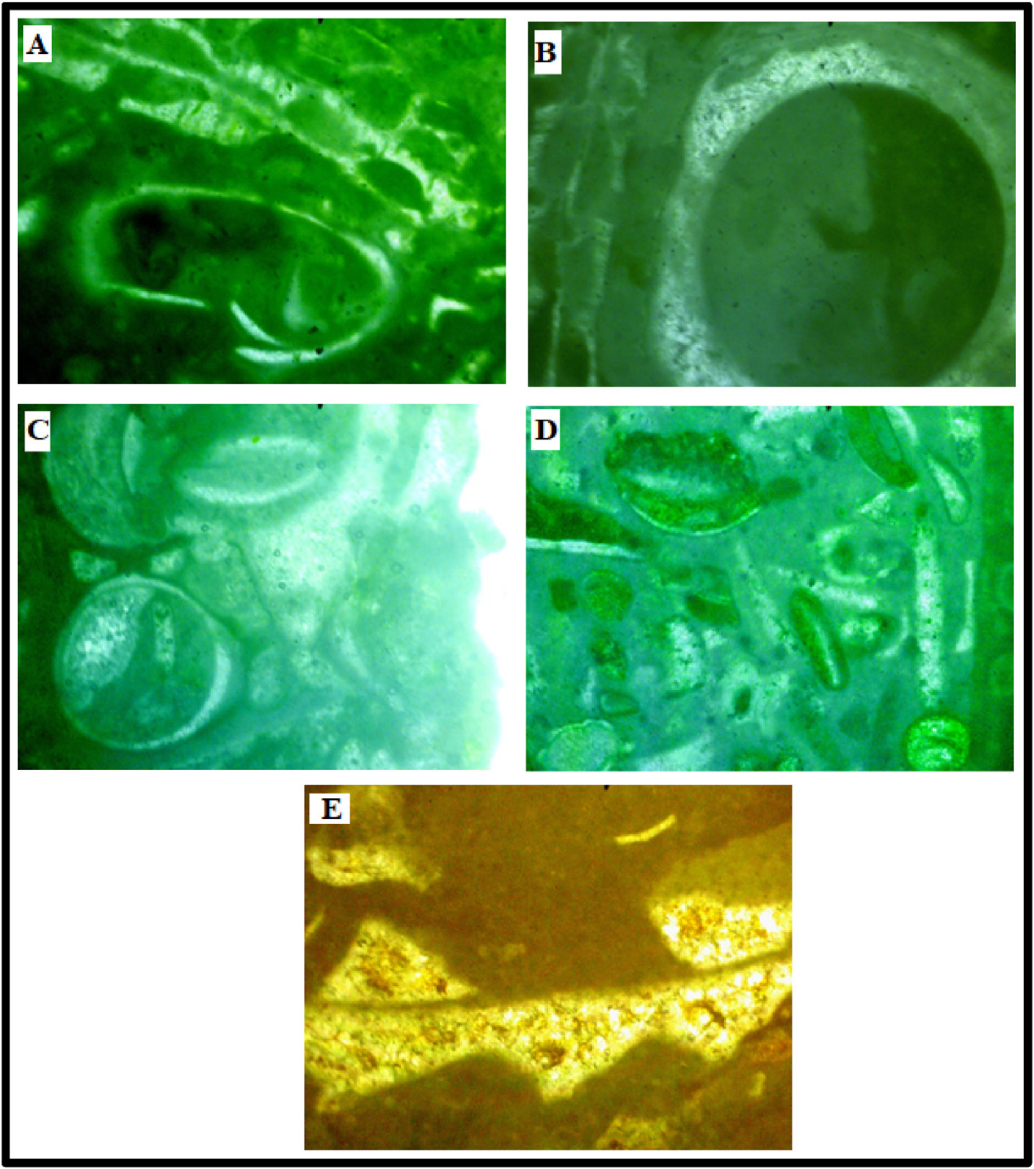
A-C. Bryozoan crinoid gastropod wackestone fenestrate bryozoans (coralline algae). The crinoids show geopetal infillings with partial mud fillings and microsparite cements. **D.** Mollusca ooid wackestone half-moon ooidal carapaces with geopetal fabric and molluscan fragments are floating in a mud matrix. **E.** Molluscan fragment with a crinkled lamella (probably brachiopod fragment).

Bryozoan-crinoid-gastropod wackestone - Fenestrate bryozoans with thick rimmed gastropod shells and isopachous microspar cement filled crinoids were observed (Figures 6A-6C). The matrix is mainly carbonate mud/silt-sized particles. The crinoids show geopetal structure in some places with precipitated spar cement and bioclastic material-infillings (Figure 6B). Isopachous envelopes of sparry calcite can be seen around the bioclasts.

Molluscan ooid wackestone - The bioclastic wackestone contains fragments of fairly thick molluscs (Figures 6E and 6F) and some half-moon non-skeletal grains. The interparticle pore spaces have been filled/separated by micritic cement, the molluscan remains have be calcified. The ooids seems distorted or broken in some grains and there were brachiopod remains with crinkled lamella (Figure 6F).

### 3.2 X-ray diffraction (XRD)

**Figure 7:**
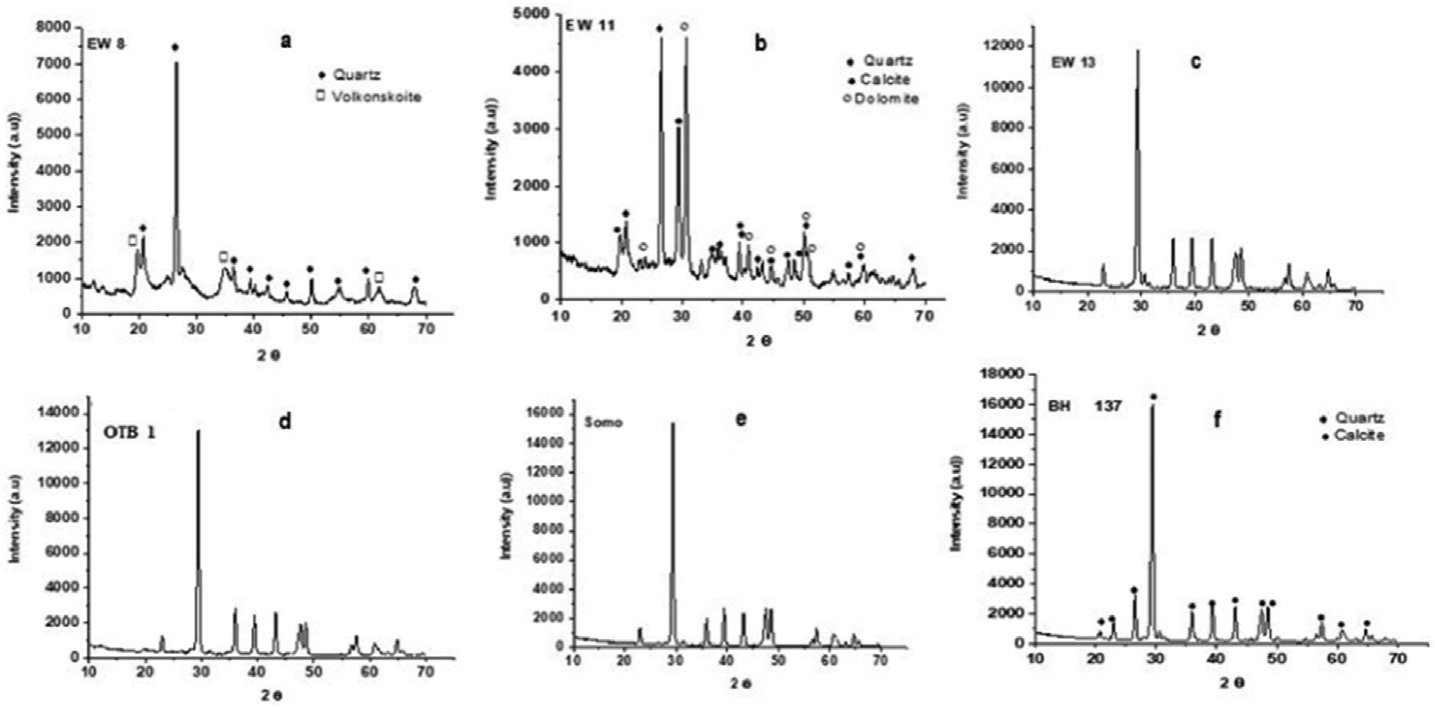
X-Ray Diffraction signatures of the Paleocene-Eocene (Ewekoro) Limestone. a) EW 8. showing quartz and Volkonskoite as its compositions. b) EW 11 composed of quartz, calcite and dolomite c) – e) EW 13, OTB 1 and SOMO samples all showing similar. signatures with a single peak, identified as calcite. f) BH 10 showing calcite and quartz.

The X-ray diffraction results show that the OTB and SOMO samples are composed of mainly calcite with fairly similar XRD signatures (Figure 7; Table 1). The subsurface samples also indicate calcite as the main mineralogical component and quartz in some samples (BH 10) with the exception of the sample EW 8 and EW 11, the former having volkonskoite (weathering product of serpentine) and quartz as its mineral compositions and the latter has dolomitic minerology co-occurring with calcite and quartz**.**

**Table 1:** Approximate percentage mineral compositions of limestones from Benin basin.

SAMPLE	M			
	Quartz (%)	Calcite (%)	Dolomite (%)	Volkonskoite (%)
SURFACE LIMESTONE SAMPLES				
OTB 1	-	100	-	-
OTB 2	-	100	-	-
OTB 3	-	100	-	-
OTB 4	-	100	-	-
OTB 6	-	100	-	-
SOMO	-	100	-	-
EW and BH SAMPLES				
EW 8	75	-	-	25
EW 11	28	30	42	-
EW 13	-	100	-	-
EW 16	-	100	-	-
BH 137	9	91	-	-

M – Mineral.

### 3.3 Stable isotope geochemistry

The δ^13^C values vary from -1.4 ‰, to +1.9 ‰ V_PDB_, most of the outcrop samples recorded negative values whilst the core samples have positive values (Table 2). Four samples from the upper part of EW core did not show any peaks (there were no appreciable isotopic measurements). The average value of -0.3‰ V_PDB_ [0.3 ± 1.7‰] was recorded, which is close to zero and comparable to δ^13^C values of modern carbonates sediments. The δ^13^C_(VPDB)_ values for marine carbonate rocks are usually constant and close to zero, while on the other hand, fresh-water limestones are usually enriched in ^12^C as a result of the organic influences and the δ^13^C_(VPDB)_ values are more scattered (Hudson, 1977; Hammarlund et al., 1997; Moore, 2001).

**Table 2:** Stable Isotope Results, Estimated Palaeotemperatures and the measured concentrations of manganese (Mn) and strontium (Sr) (core samples in red and outcrop samples in black).

Sample	δ^18^O_VPDB_	δ^18^C_VPDB_	δ^18^O_(VSMOW)_	Z	T^O^C	Mn (ppm)	Sr (ppm)	Mn/Sr
OTB 1	0.5	1.9	31.58	131	19.12	-	-	-
OTB 2	-2.9	-0.1	27.12	126	35.5	-	-	-
OTB 4	-2.3	-1.2	27.91	124	32.44	180	519	0.35
OTB 5	-2.5	-1.1	27.65	124	33.46	-	-	-
OTB 6	-2	0.1	28.3	127	30.94	-	-	-
EW 11	-3.5	-1.4	26.34	123	38.64	-	-	-
EW 12	-0.9	0.4	29.74	128	25.58	-	-	-
EW 14	-1.3	0.8	29.22	128	27.5	550	352	1.56
EW 16	-2.4	0.3	27.78	127	32.95	-	-	-
BH 10	-2.1	0.8	28.17	128	31.44	151	371	0.41
SOMO	-2.7	-0.2	27.39	126	34.48	349	324	1.08

In this present study, δ^18^O values range from -0.9‰ to -3.5‰ V_PDB_ (with exception of sample OTB1 which is 0.5‰ V_PDB_) and an average δ^18^O value of -2.01‰ V_PDB_ [-2.0 ± 1.5‰ V_PDB_]. Sample EW-11 was identified as an outlier with 99% confidence level (Table 2).

### 3.4 Depositional environment and paleotemperature deduced from δ^18^O and δ^13^C

One of the best discriminations between marine and freshwater limestones is given by Eq. (1) (Keith and Weber, 1964).

(1) Z = a (δ^13^C + 50) + b (δ^18^O + 50)

**Where,** a = 2.048, b = 0.498,

δ^18^O = Oxygen isotopic composition of the limestone (v_PDB_).

δ^13^C = Carbon isotopic composition of limestone (v_PDB_).

Limestone samples with a Z value above 120 would be classified as marine, while those with Z value below 120 as fresh-water limestone. The studied Paleocene-Eocene (Ewekoro) Limestone samples in the study area are classify into marine limestone with calculated Z values ranging from 123 to 131.

Estimated paleotemperature values for the limestone ranging from 19 °C to 39 °C (Table 2) was inferred using Eq. (2) (Shackleton and Kennett, 1975).

(2) T = 16.9–4.38(δc^18^O – δw^18^O) + 0.1(δc^18^O – δw^18^O)^2^

**Where,** δc^18^O = Oxygen isotopic composition of limestone (v_PDB_).

δw^18^O = Oxygen isotopic composition of seawater (vs_MOW_), (which is assumed to be dw^18^O = δ^18^O_(VSMOW)_ -1.00%; Shackleton and Kennett, 1975; Kim et al., 2015).

This equation is an expression of the isotopic equilibrium between water and calcite.

**Figure 8:**
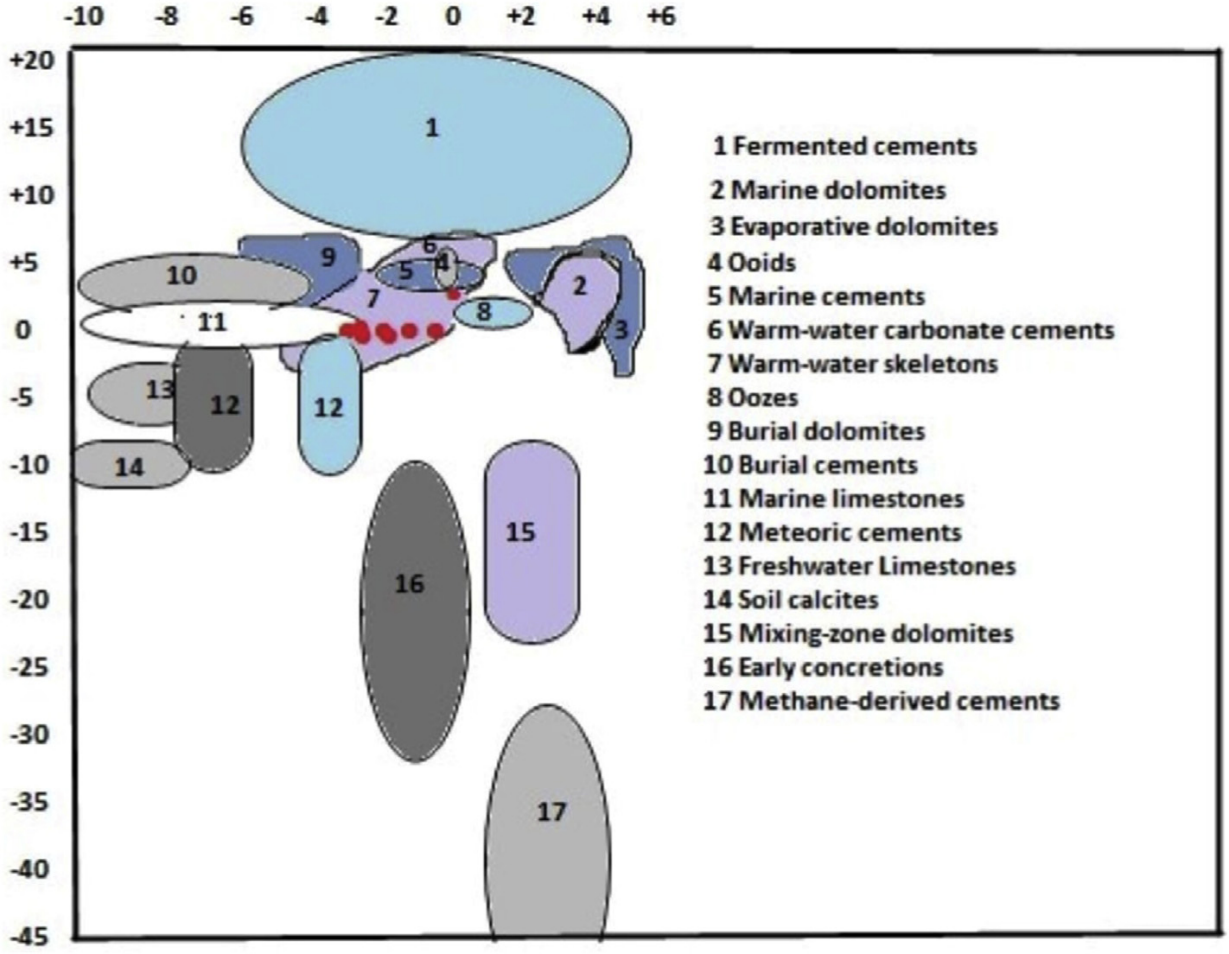
Position plots of the studied samples on the δ^18^O versus δ^13^C bivariate diagram. The studied samples plot within the warm-water skeletons and marine cement fields. (After Nelson and Smith, 1996; Used with permission from Taylor and Francis, New Zealand Journal of Geology & Geophysics).

A wide-ranging field of isotopic signatures of carbonates was proposed by Hudson (1977), this diagram is a bivariate plot of δ^18^O versus δ^13^C (Figure 8), which was modified by Nelson and Smith (1996) to include fields for carbonate components, sediments, limestones, concretions, dolomites and cements. All the studied samples plot in the warm-water skeleton and marine limestone fields while only one sample plot at the edge of the meteoric cements field (Figure 8).

### 3.5 Rare earth element (REE)

**Figure 9:**
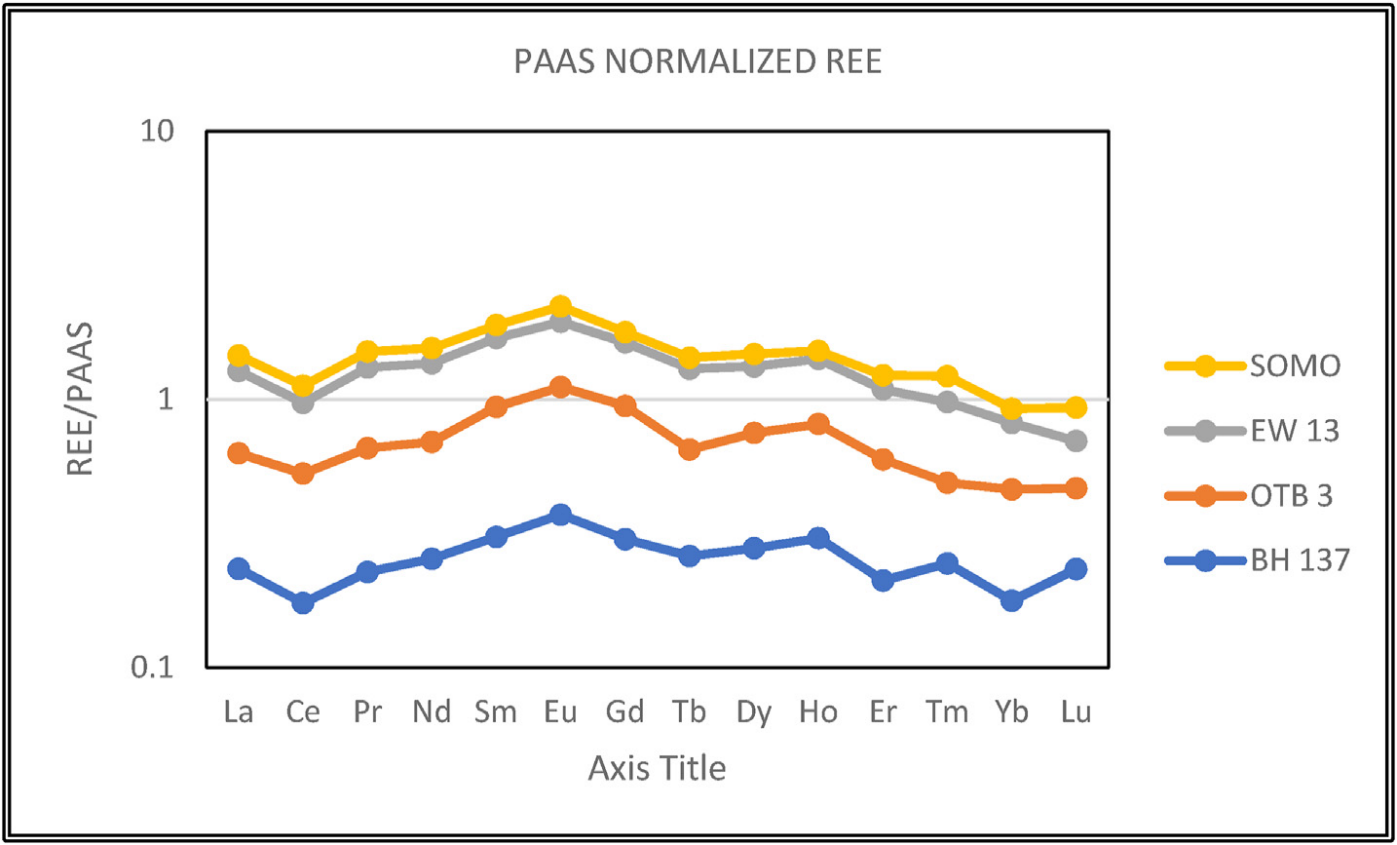
PAAS normalized REE diagram for the Paleocene-Eocene Ewekoro limestone.

The total REE (ƩREE) of the Paleocene-Eocene Ewekoro limestone ranged from 39.93 to 103.11 ppm (parts per million; Table 3). Negative cerium anomaly (Ce/Ce∗) which was calculated using Eq. (3) (German and Elderfield, 1990), showed depletion in cerium (0.67–0.86, Table 3) relative to its neighbouring light rare earth elements (LREE). The Post Archean Australian Shale (PAAS; Mclennan, 1989) normalized ratio of LREEs (La - Sm) to Heavy REEs (HREE, Gd–Lu), showed that there was enrichment of LREEs (Nd_SN_/Yb_SN_ 1.43 to 1.89; _SN_ – Shale Normalized; Table 3, Figure 9). Likewise, the (La/Yb)_SN_ values range from 1.31 to 1.82, (Dy/Yb)_SN_ range from 1.40 to 1.66 and the evaluated Er/Nd values in the Paleocene-Eocene Ewekoro limestone are quite low (0.06–0.07). All these indicates preferential scavenging process of LREE from the environment of the limestones during post depositional processes (Nagendra et al., 2011) and points to detrital contaminations (Nagendra et al., 2011; Akaegbobi and Ogungbesan, 2016; Srivastava and Singh, 2019).

**Table 3:** Rare earth element concentrations in the studied Ewekoro limestones.

SAMPLE	BH 1	OTB 3	EW 13	SOMO
La (ppm)	8.9	15.1	24.7	7
Ce (ppm)	13.83	28.2	35.11	12.3
Pr (ppm)	2	3.8	5.8	1.7
Nd (ppm)	8.6	14.9	22.7	6.4
Sm (ppm)	1.7	3.5	4.2	1.1
Eu (ppm)	0.4	0.8	0.9	0.3
Gd (ppm)	1.4	3	3.2	0.7
Tb (ppm)	0.2	0.3	0.5	0.1
Dy (ppm)	1.3	2.2	2.7	0.7
Ho(ppm)	0.3	0.5	0.6	0.1
Er (ppm)	0.6	1.1	1.4	0.4
Tm (ppm)	0.1	0.1	0.2	0.1
Yb(ppm)	0.5	0.8	1	0.3
Lu (ppm)	0.1	0.1	0.1	0.1
ƩREE	39.93	74.4	103.11	31.5
(La/Yb)_SN_	1.31	1.39	1.82	1.72
(La/Nd)_SN_	0.92	0.90	0.97	0.97
(Nd/Yb)_SN_	1.43	1.55	1.89	1.77
(Dy/Yb)_SN_	1.57	1.66	1.63	1.41
Er/Nd	0.07	0.07	0.06	0.06
Eu/Eu∗	0.48	0.66	0.69	0.47
Ce/Ce∗	0.72	0.86	0.67	0.84
Pr/Pr∗	1.06	1.08	1.18	1.12

**Figure 10:**
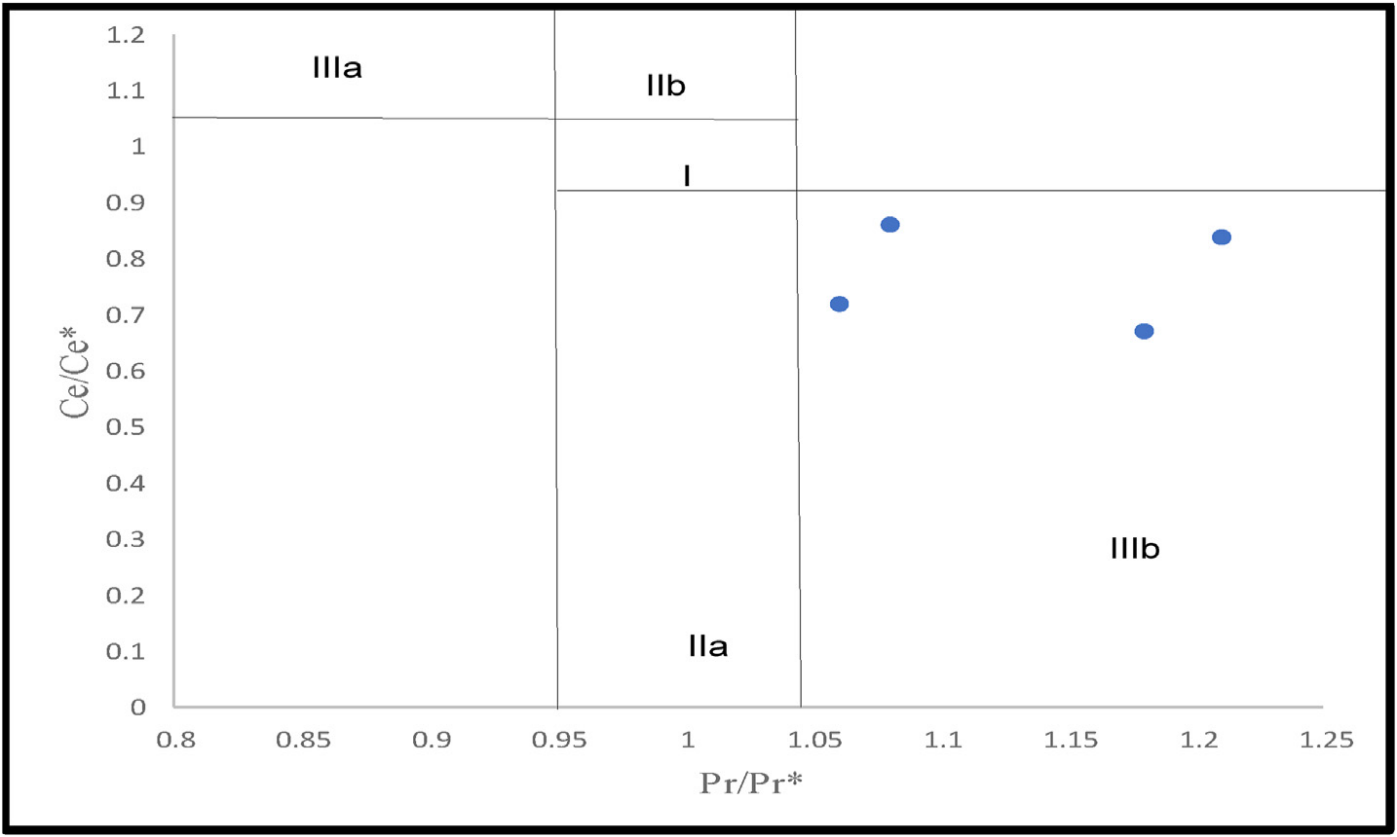
A plot of PASS normalized Ce/Ce∗ against Pr/Pr∗. Field I: no anomaly; Field IIa: positive La anomaly causes apparent negative Ce anomaly; Field IIb: negative La anomaly causes apparent positive Ce anomaly; Field IIIa: real positive Ce anomaly; Field IIIb: real negative Ce anomaly. The studied samples fall within IIIb, indicating genuine Ce anomaly. (After Bau and Dulski, 1996; Used with permission from Elsevier, Precambrian Research).

Bau and Dulski (1996) suggested that elevated La_SN_ values can give erroneous Ce/Ce∗ values, this error can be mitigated by estimating the Pr/Pr∗ value using Eq. (4) (Bau and Dulski, 1996) and plotting it against the Ce anomaly, to identify the La anomalies. According to the proposal, samples plotting in regions IIIa and IIIb are actual positive and negative Ce anomalies respectively. The studied limestones all plotted in the real negative region (Figure 10), indicating that they were unaffected by excessive La _SN_.

(3) **Ce/Ce∗ = 3Ce**_**SN**_**/ (2La**_**SN**_**+ Nd**_**SN**_**)**

(4) **Pr/Pr∗ = Pr**_**SN**_**/ (0.5Ce**_**SN**_**+ 0.5Nd**_**SN**_**)**

(5) **Eu/Eu∗ = Eu/ (Sm + Gd)**^**0.5**^

## 4 Discussion

### 4.1 Depositional environment

The studied samples are mainly composed of calcite and quartz, with one dolomitic sample and another volkonskoite-bearing sample. The microfacies revealed by microscopic study of the thin sections display different depositional environments, from vadose meteoric to shallow marine deposition environments and varying diagenetic alterations, mineral replacement, micritization, dolomitization and stylolitization.

Post depositional overprints are visible with the replacement of originally aragonitic shell of the molluscan fragments by coarse-grained calcite crystals (the coarse grain cements are indicative of a high energy environment (El-Ghali et al., 2013). The first whorls are completely obliterated and the whorls exhibit various cement generations or are infilled by micritic sediment. The neomorphic mosaic-like microtexture, equant grains of calcite, caused by the dissolution of former aragonite needles, is also a pointer to post deposition alteration. The observed presence of partial infilling (geopetal infillings fabrics) indicates the water level in the cavity at the time of deposition of the internal sediment’ (Flugel, 2010). The geopetal feature is commonly indicative of reworking and redeposition and also associated with deposition in vadose zones (Flugel, op cit.). Generally, meniscus cementing observed in some of the slides usually round off pore spaces and are typical of meteoric-vadose zones (Hartmann and Beaumont, 1999). The presence of carbonate mud/fine grained micritic material is indicative of a low energy environment or maybe due to the association of Paleocene-Eocene (Ewekoro) limestone with shaly interbeds; and the isopachous micritic envelopes observed around the outer lamella of the some of the bioclast is as a result of microbial attack/borings (Reid and MacIntyre, 2000; Oluwajana et al.,2020). The radial fibrous calcite of the calcareous algae are common features in meteoric vadose and phreatic environments (Flugel, 2010).

The preponderance of many skeletal fragments of bivalves, cephalopods, gastropods, pelecypods and foraminifera are indicative of marine deposition probably in an open system, incompleteness of some fossils might be due to high energy and rapid burial. Stylolitization observed in some slides are effects of mechanical and chemical processes of compaction (Adams and Mckenzie, 1998).

The carbon isotopic values of the studied limestones are similar those obtained for marine limestones. The δ^13^C values showed some marked variations as the outcrop samples recorded negative δ^13^C while core samples did not show carbon depletion. In general, the limestone collected close to the subaerial exposure surface (which might have induced meteoric diagenetic alterations) has more negative δ^13^C values than the limestone deposited several depths below the surface (Allan and Matthews, 1982; Madhavaraju et al., 2013). High algal population and photosynthetic activity in the shallow marginal marine environment can give positive δ^13^C values (Armstrong-Altrin et al., 2011; Hossain et al., 2013), alternatively, the presence of organic remains can be responsible for the negative values (Armstrong-Altrin et al., 2011). Therefore, the limestones were interpreted as shallow marginal marine in samples with positive δ^13^C values. While the samples with negative δ^13^C values are probably due to breakdown in oceanic productivity as outlined by Marshall (1992) at the Cretaceous – Paleogene boundary or an enhanced supply of isotopically light, organically derived carbon into the carbon cycle through regression and erosion (Nagarajan et al., 2013). The recorded carbon isotopes in the studied samples did not reveal extreme alteration as they were only slightly negative (most depleted being sample EW11, with a δ^13^C of -1.4‰), because post deposition processes do not alter carbon isotope significantly (Madhavaraju et al., 2013).

Shale-normalised, seawater-type REE patterns are characteristically enriched in HREE (Bi et al., 2019) and a depletion of cerium (Ce) with respect to its REE neighbours, lanthanum (La). and praesodymium (Pr)., or neodymium (Nd) (German and Elderfield, 1990; Shields and Stille, 2001). Even though, the shale normalized pattern of the studied limestones (Figure 9), shows Ce_SN_ depletion relative to the neighbouring LREE, the PAAS normalized LREE/HREE showed enrichment of LREEs, this observed REE signatures are characteristic of sediments with terrigenous influence (Madhavaraju and Lee, 2009; Akaegbobi and Ogungbesan, 2016). The negative Ce anomaly can be associated with oxidation common in shallow marine environments (German and Elderfield, 1990; Shields and Stille, 2001; Madhavaraju and Lee, 2009; Nton and Adeyemi, 2015; Singh et al., 2016). The evaluated (Dy/Yb)_SN_ ratios are similar to the Kudankulam limestones (Armstrong-Altrin et al., 2003) but these values are higher than that of modern seawater which ranges from ∼0.8–1.1 (Nagendra et al., 2011; Singh et al., 2016).

### 4.2 Post- deposition overprints

The range of values of δ^18^O shows that the samples all categorise into the marine limestone, with values similar to those obtained by Veizer (1983). Cuna et al. (2001) indicated that δ^18^O of freshwater carbonate rocks are usually depleted as compared to sea waters. Negative values of δ^18^O are an indication of meteoric water diagenesis (Armstrong-Altrin et al., 2011). Diagenesis often results in negative δ^18^O values in marine carbonates, because cementation and re-crystallization often takes place in fluids depleted in δ^18^O with respect to sea water (e.g meteoric water) or at elevated temperatures (burial conditions) (Marshall, 1992; Armstrong-Altrin et al., 2011). Decreasing δ^18^O value is connected with decreasing salinity and increasing temperatures (Hudson, 1977). Negative values of δ^18^O in many limestones is supportive of cementation under burial and/or meteoric conditions (Armstrong-Altrin, 2009; Madhavaraju et al., 2013).

**Figure 11:**
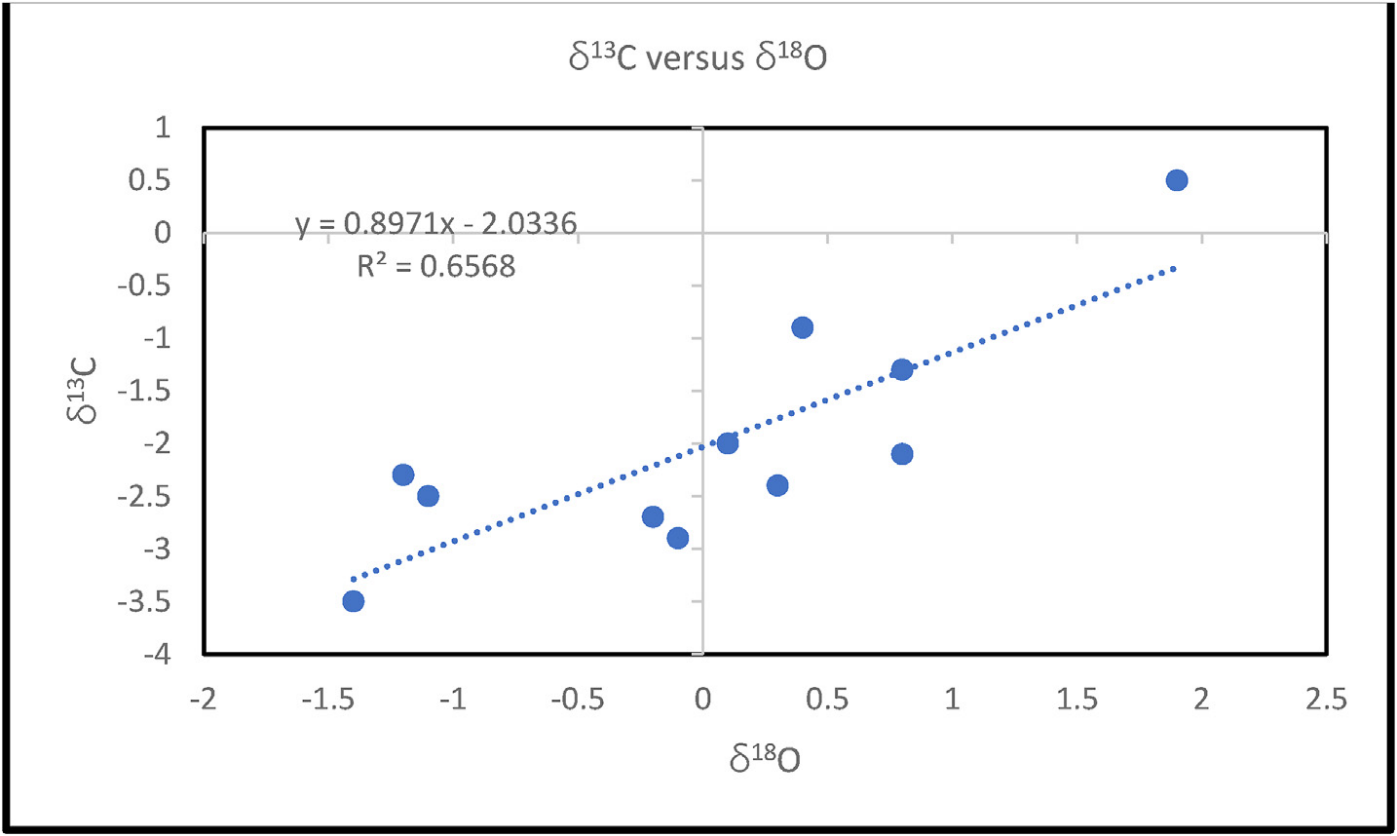
δ^13^C versus δ^18^O bivariate plot for the Paleocene-Eocene (Ewekoro) Limestone. The samples show a positive correlation, R^2^ = 0.66, n = 4. Which is indicative of post deposition overprint on the isotopic values.

The intensity of diagenetic alteration in limestones is estimated by plotting δ^13^Cversus δ^18^O values (Figure 11). The δ^13^C values show statistically positive correlation with δ^18^O values (R^2^ = 0.66, n = 11), such positive relationship between δ^13^C and δ^18^O might be an indication that the Paleocene-Eocene (Ewekoro) limestone were altered by diagenesis (Marshall, 1992; Armstrong-Altrin et al., 2011). However, studies focused on the early Paleogene suggested a period of global warming, which started in the mid Paleocene, through the Paleocene – Eocene transition (Paleocene – Eocene Thermal Maximum, PETM) at ∼ 56Ma, and culminated during the Early Eocene Climatic Optimum (EECO), between ∼53Ma to 51-50Ma (Zachos et al., 2010; Slotnick et al., 2015; Patra et al., 2021). These events decreased salinity and lowered δ^18^O of the oceans, as melting ices resulted in global sea level rise. These phenomena would account for the low δ^18^O in the studied samples as previous work in the basin also recorded evidence of warming in the early Paleogene sediments (Adeonipekun, and Oyelami, 2015; Frieling et al., 2017). The relatively depleted δ^18^O and the anomalous carbon isotopic excursion of sample EW-11 is associated with the PETM, and the estimated temperature of ∼39 °C is consistent with temperature suggestions of Frieling et al. (2017). It is obvious from the XRD results that EW-8 is not calcitic and this might be the reason why it did not give peak δ^18^ O results. The upper 8m of the EW-1 core could be the Akinbo Formation which has been reported to be glauconitic (Nwajide, 2013).

**Figure 12:**
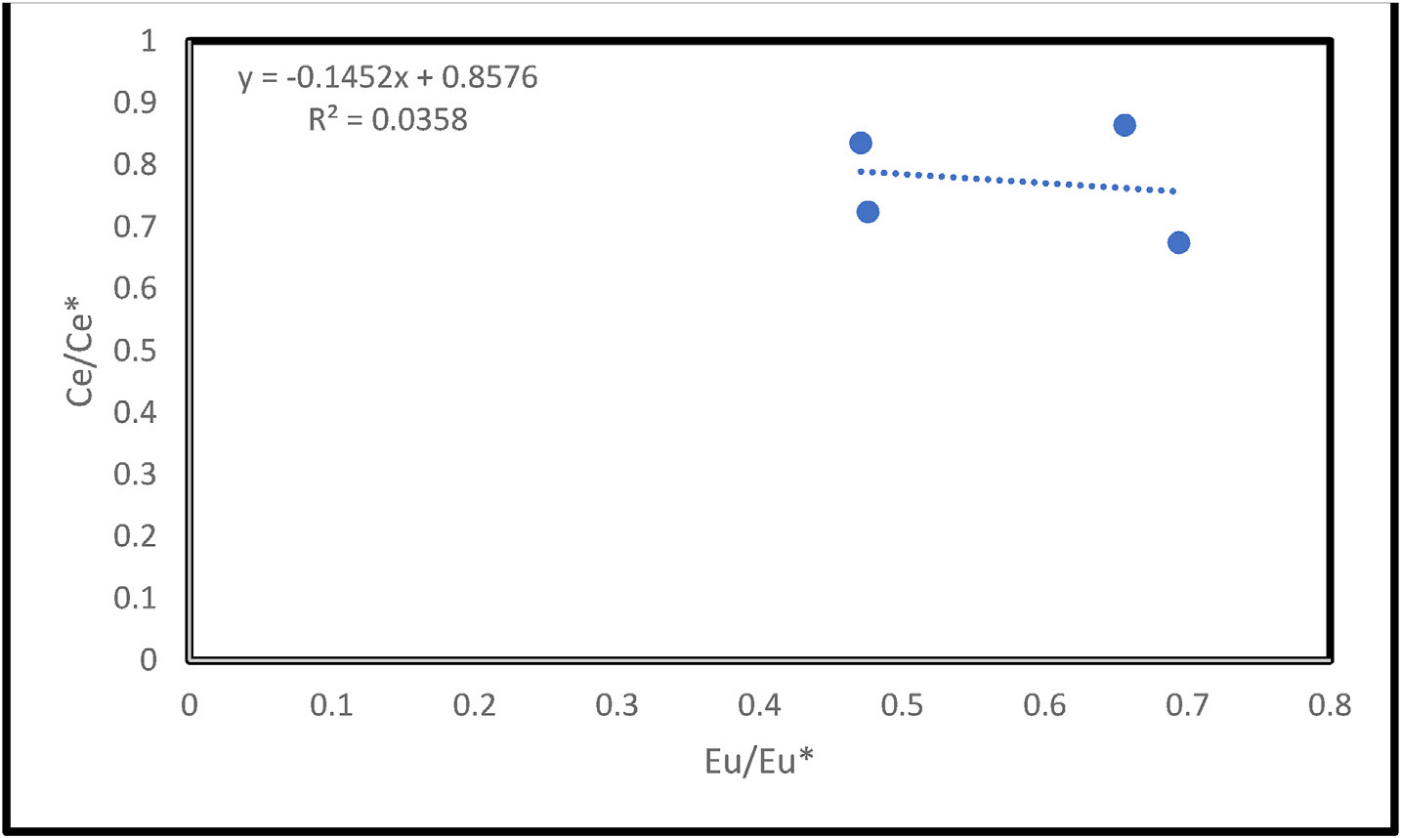
Ce/Ce∗ vs Eu/Eu∗ plot shows that there is no correlation between the Ce anomaly and the Eu anomaly, R^2^ = 0.04, n = 4. This underplays the effect of diagenesis on the REE distribution.

Er/Nd ratio in normal seawater is about 0.27 (Bau and Dulski, 1996; Shields and Stille, 2001; Singh et al., 2016). The preferential concentration of Nd relative to Er can reduce the Er/Nd value to <0.1 and this might be due to the effect of detrital input and post deposition overprint (German and Elderfield, 1990). The low Er/Nd values recorded in the studied samples has been attributed to detrital input, with evidence from the XRD, (which shows quartz as one of its components of some samples), as well as post deposition overprints, observed in the studied petrographic slides. Eu anomaly (Eu/Eu∗) values can aid in the interpretation of the geochemical condition of the depositional environments of limestones (Abedini and Calagari, 2015). Eu/Eu∗ values, computed using Eq. (5) (Abedini and Calagari, 2015), for the Ewekoro limestones showed extreme depletion with values 0.48, 0.66, 0.70 and 0.47 for samples BH 137, OTB 3, EW 13 and SOMO samples respectively (Table 3) with a mean value of 0.58. These values are similar to the Eu/Eu∗ values of Limestones of the Kallankurichchi Formation (Madhavaraju and Ramasamy, 1999) and the Kudankulam carbonates (Armstrong-Altrin et al., 2003). Murray et al.,1991 opined that Eu < 1 can be due to aeolian inputs and Eu > 1 was attributed to hydrothermal influence. So also, Eu/Eu∗ anomalies can be due to diagenesis (Murray et al., 1991; Abedini and Calagari, 2015; Ozkan, 2019). The negative Eu anomalous behaviour of the studied samples have therefore been attributed to post depositional overprinting as no aeolian inputs have been reported in the Paleocene- Eocene Ewekoro limestone. A strong positive correlation between Eu/Eu∗ and Ce/Ce∗ can be used as a measure of post deposition overprints on sediments (Abedini and Calagari, 2015). The evaluated correlation between Eu/Eu∗ and Ce/Ce∗ in the studied limestone is negative (R^2^ = 0.04, n = 4, Figure 12). This negates the assumption that diagenesis played a major role in the distribution of the REEs.

The Mn/Sr ratio is also useful for the assessment of post-depositional changes in the carbonate rocks (Veizer, 1983; Kaufman and Knoll, 1995; Marshall, 1992; Madhavaraju et al., 2013; Nagarajan et al., 2013). The measured concentrations of Mn for samples OTB 4, EW 14, BH10 and SOMO were 180,550, 151 and 349 ppm respectively. The recorded Sr concentrations are 519, 352, 371 and 324ppm respectively, with the calculated corresponding ratios of (Mn/Sr) 0.35, 1.56, 0.41 and 1.08 respectively (Table 2). Limestones with Mn/Sr ratios less than 2 indicate that they were not subjected to significant diagenesis (Jacobsen and Kaufman, 1999; Nagarajan et al., 2008). The observed Mn/Sr ratios in the Ewekoro limestones indicate that they were only slightly altered with some values above 1 but less than 2.

## 5 Conclusions

Three microfacies were inferred from the petrographic study of the Paleocene-Eocene Ewekoro limestone: wackestone, packstone and grainstone. The limestones were deposited in shallow marine environment with δ^13^C values close to that of modern marine carbonates and Z values above 120. The presence of coarse grained neomorphic calcite cements indicate a high energy environment, while micritic deposits in the wackestone indicate low energy environment. The estimated palaeotemperature shows that the limestones were deposited in a warm climate (tropical). The carbon isotopic results indicate reduced oceanic productivity identified globally at the Cretaceous to Paleogene boundary. Although, the petrography revealed significant evidence of post deposition overprints (dolomitization, compaction, micritization), the Mn/Sr ratio indicates that the diagenetic alterations have not overprinted the primary isotopic values. The low oxygen isotopic signals were associated with the warming events that occurred in the early Paleogene and the PETM was recognized in sample EW11. The REE signatures connote terrigenous contamination and the negative Ce/Ce∗ values indicate an oxidizing environment of deposition.

## Declarations

### Author contribution statement

Adelabu, I.O.: Conceived and designed the experiments; Performed the experiments; Analyzed and interpreted the data; Contributed reagents, materials, analysis tools or data; Wrote the paper.

Opeloye, S.A.: Conceived and designed the experiments; Contributed reagents, materials, analysis tools or data.

Oluwajana, O. A: Analyzed and interpreted the data; Contributed reagents, materials, analysis tools or data.

### Funding statement

This research did not receive any specific grant from funding agencies in the public, commercial, or not-for-profit sectors.

### Data availability statement

Data included in article/supplementary material/referenced in article.

### Declaration of interests statement

The authors declare no conflict of interest.

### Additional information

No additional information is available for this paper.
